# Effect of chronic vapor nicotine exposure on affective and cognitive behavior in male mice

**DOI:** 10.1038/s41598-024-56766-z

**Published:** 2024-03-19

**Authors:** Laura B. Murdaugh, Cristina Miliano, Irene Chen, Christine L. Faunce, Luis A. Natividad, Ann M. Gregus, Matthew W. Buczynski

**Affiliations:** 1 School of Neuroscience, Virginia Polytechnic Institute and State University, 970 Washington St SW, Life Sciences I, Blacksburg, VA 24061 USA; 2 Translational Biology, Medicine, and Health, Virginia Polytechnic Institute and State University, Blacksburg, VA USA; 3 Department of Chemistry, Virginia Polytechnic Institute and State University, Blacksburg, VA USA; 4 College of Pharmacy, Division of Pharmacology and Toxicology, University of Texas at Austin, Austin, TX USA

**Keywords:** ENDS, e-cigarette, Nicotine, Vaping, Cognitive, Addiction, Neuroscience, Physiology

## Abstract

Nicotine use is a leading cause of preventable deaths worldwide, and most of those who attempt to quit will relapse. While electronic cigarettes and other electronic nicotine delivery systems (ENDS) were presented as a safer alternative to traditional cigarettes and promoted as devices to help traditional tobacco smokers reduce or quit smoking, they have instead contributed to increasing nicotine use among youths. Despite this, ENDS also represent a useful tool to create novel preclinical animal models of nicotine exposure that more accurately represent human nicotine use. In this study, we validated a chronic, intermittent, ENDS-based passive vapor exposure model in mice, and then measured changes in multiple behaviors related to nicotine abstinence. First, we performed a behavioral dose curve to investigate the effects of different nicotine inter-vape intervals on various measures including body weight, locomotor activity, and pain hypersensitivity. Next, we performed a pharmacokinetic study to measure plasma levels of nicotine and cotinine following chronic exposure for each inter-vape interval. Finally, we utilized a behavior test battery at a single dosing regimen that produces blood levels equivalent to human smokers in order to characterize the effects of chronic nicotine, vehicle, or passive airflow and identified nicotine-induced impairments in cognitive behavior.

## Introduction

While a half-century of effort has successfully reduced overall nicotine use from its highest levels, 18.7% of US adults^1^ and 10.0% of US middle- and high-school youths^2^ report current tobacco use. Despite progress in reducing their consumption, tobacco and nicotine use remains a leading cause of preventable death in the US. Since the advent of electronic cigarettes (e-cigarettes) and electronic nicotine delivery systems (ENDS), many users have shifted away from using traditional combustible cigarettes toward these novel methods of nicotine delivery^3,4^. ENDS are small battery-powered devices that heat and deliver a mixture of a liquid vehicle, drug (*e.g.*, nicotine, Δ9-tetrahydrocannabinol), and/or flavors for user inhalation^5^. Despite the popularity of such devices, there is limited basic or preclinical research^6^ into the physiological, behavioral, and cognitive effects of chronic vapor-based exposure (CVE) to nicotine as compared to traditional cigarettes.

The use of animal models allows researchers to perform in-depth investigations of the mechanisms and/or circuitry underlying the behavioral and cognitive effects of chronic vapor-based nicotine exposure, which is still under-studied. Historically, research on the health implications of chronic nicotine exposure^7–9^ has employed non-inhalation routes of administration (*e.g.*, intravenous self-administration, continuous subcutaneous exposure) to model human nicotine consumption. It is well known that the route of administration strongly influences the subjective or reinforcing effects of a drug, ultimately contributing to its addictive potential^10–12^. For these reasons, intravenous self-administration of nicotine has considerable face validity^13,14^ as the drug quickly crosses the blood–brain barrier to exert central nervous system effects. However, the development of ENDS allows the study of nicotine effects by using the same route of administration of clinical populations. While some researchers have begun to employ ENDS-based vapor delivery systems to study the neurobiological effects of chronic nicotine exposure^15–17^ or other substances^18^, most studies to date have investigated a limited number of dosing regimens or behavioral outputs.

To address this gap, we first investigated the dose-dependent physical, behavioral, and pharmacokinetic effects of nicotine under chronic exposure conditions. Based on these experiments, we selected the chronic nicotine dosing procedure that produced pharmacokinetic and behavioral responses consistent with those observed in human smokers, and further analyzed performance on a multi-faceted test battery of affective and cognitive behaviors.

## Materials and methods

### Animals

C57BL/6J male mice (8–10 weeks at the beginning of experiments) were obtained from Jackson labs and group housed five to a cage on a 12-h reverse light cycle (21:00 on/9:00 off). Animals were given ad libitum access to standard chow and water, except when otherwise stated. Chronic Vapor Exposure and all behavioral experiments were performed during the dark cycle unless otherwise stated. All experimental procedures were conducted in accordance with the guidelines for the care and use of animals, as set by the National Institutes of Health. Protocols were approved by the Institutional Animal Care and Use Committee (IACUC) of Virginia Tech (Blacksburg, VA, USA), and studies conformed to ARRIVE guidelines.

### Chronic vapor exposure (CVE) paradigm

CVE was performed using a vacuum-based vapor exposure system from La Jolla Alcohol Research^17,19,20^. Animals were placed into sealed home cages with their standard housing cage-mates for 8 h a day, 5 days a week (9:00–17:00 M-F) before being returned to their standard home cages. The sealed cages continuously received clean room air from a down-stream vacuum pump (16 L/m), and a computer-controlled Electronic Nicotine Delivery System (ENDS) delivered vapor (3 s, 200 °C) at pre-defined inter-vape intervals (2, 5, 10, 15, and 60 min). The e-liquid vehicle (VEH) consisted of 50% propylene glycol (Sigma-Aldrich, P4347) and 50% vegetable glycerin (Sigma-Aldrich, G5516) (50/50 PGVG), and all experiments with nicotine (NIC) used a dose of 20 mg/ml (−)−nicotine (Sigma Aldrich, N3876). During all vapor exposure procedures, mice could move freely within the home cage with ad libitum access to food and water.

### Optimization of the CVE paradigm

To determine optimal conditions for the CVE paradigm we utilized 5 cohorts of mice exposed to one of 5 different nicotine vapor inter-vape intervals (2, 5, 10, 15, or 60 min) or air control for multiple weeks. Behavioral tests were performed after week 3.

#### Changes in body weight

Mice were weighed once per week on Mondays, immediately after vapor exposure.

#### Locomotor activity

The locomotor activity of mice was recorded in clear cages by using a video tracking system (AnyMaze) to measure the total distance traveled (m) for 1 h prior to starting the vaping session and 1 h immediately following the vaping session.

#### Tactile allodynia

We performed a time course at baseline and 1, 2, 4, 16, and 24 h after vapor exposure; tactile allodynia was evaluated using manual von Frey filaments with buckling forces between 0.02 and 2 g (Touch Test, Stoelting Co.) applied to the mid-plantar surface of each hind paw using the up-down method as we have described previously^21–23^. All testing was performed under red light during the dark cycle, and the researcher was blinded to the experimental conditions. Any mouse with a basal 50% paw withdrawal threshold (PWT) ≤ 0.79 g was excluded from the study. For all measurements, PWTs from both hind paws were averaged. Data were expressed as 50% gram thresholds vs time.

#### Pharmacokinetic analysis

Submandibular blood sampling was performed as previously described^24,25^ by collecting blood in EDTA-coated tubes at different time points (5 min or 1, 2, 4, and 24 h) following chronic vaping exposure. Blood samples were immediately centrifuged at 3000×*g* for 15 min at 4 °C and plasma was stored at − 80 °C until analysis. The samples were processed and analyzed by using UPLC-MS/MS. The nicotine extraction was performed by adding 5μL of isotopically labeled nicotine-d4 and cotinine-d3 internal standards to 50 μl of plasma, followed by 100 μL Brine NaOH solution and 100 μL of Methyl tert-butyl ether (MTBE). After briefly vortexing, samples were placed in dry ice for approximately one minute to freeze the aqueous layer^26^. The organic layer was transferred to a mass spec vial and analyzed using a 1290 Infinity II LC System (Agilent Technologies) coupled with a 6495 triple quadrupole mass detector (Agilent Technologies). The chromatographic separation was performed with an Acquity UPLC^®^ BEH HILIC column (Waters Corporation, 2.1 mm I.D. × 100 mm, particle size 1.7 μm) at a flow rate of 400 µL/min at 35 °C, by injecting 2µL of sample. The mobile phase consisted of solvent A (0.2% formic acid, 10 mM ammonium formate in water) and solvent B (Acetonitrile, 0.2% formic acid). The analytes were eluted with the following gradient: 0–1.50 min, 99% (B); at 2.7 min, 98% (B); at 3 min, 95% (B); at 3.1 min, 70% (B); 3.10–5.10 min, 70% (B); at 5.20 min, 40% (B); 5.20–7.50 min, 40% (B); at 7.51 min, 95% (B); at 8.50 min, 99% (B); 8.50–10 min, 99% (B). The acquisition mode used was multiple reaction monitoring (MRM) in positive mode. The following transition ions were monitored for the analytes and their isotope-labeled internal standards: Nicotine (163.2 132.4), d4-nicotine (167.2 136.4), Cotinine (177.2 80), d3-cotinine (180.2 101). A 9-point calibration curve containing Nicotine (0.78–200 ng/ml) and Cotinine (2.34–600 ng/ml) was constructed using the peak area ratios of the drugs to their deuterated internal standards. Plasma control samples were run to calculate the limit of quantification (LOQ) which was 6.6 ng/ml for Nicotine and 18.7 ng/ml for Cotinine, respectively. The limit of detection (LOD) was calculated as the amount of compound able to give a 3 times higher response than noise signal and it was 0.78 ng/ml for Nicotine and 0.3 ng/ml for Cotinine. Pharmacokinetic parameters for blood level time courses such as AUC, Cmax, and t1/2 are reported in Table 1.

**Table 1:** Pharmacokinetic parameters of Nicotine CVE (Fig. 2).

	Inter-vape interval			
**Parameter**	**5 min**^**(+)**^	**10 min**^(^*^)^	**15 min**^**(#)**^	**60 min**^**(^)**^
AUC (ng*hr/ml)	661 ± 60^####,^^^^^	488 ± 39^####,^^^^^	300 ± 14^^^^^^	135 ± 6
**Cmax (ng/ml)**	155 ± 11	83 ± 21	24 ± 3	13 ± 0.9
**t1/2 (min)**	5.8	8.1	15.1	19.8

AUC, the area under the plasma concentration–time curve from t = 0 to t = 24 h;

C_max_, maximum observed plasma concentration; t_1/2_, half-life. ****P < 0.0001 vs. VEH by two-way.

Repeated measures ANOVA followed by Tukey post-hoc. n = 7 per group.

### Behavioral assessments of nicotine CVE at 10-min inter-vape interval

#### Open field test

OFT was performed 2 h after completion of vapor exposure as we have described previously^22^. The mouse’s position was assessed by AnyMaze software using an overhead camera to measure the total distance traveled (m), time spent immobile (s), time spent in edge or center zone, and number of center entries.

#### Light/Dark box test

LDT was performed at 2 and 24 h after completion of vapor exposure as we have described previously^22^. Time spent on either side (s), latency to exit from the dark chamber (s), and average dark visits (s) were recorded.

#### Splash test

The Splash test protocol was conducted as we described previously^22^ 2 h after the last vapor exposure using tap water. The total time spent grooming was recorded.

#### Sucrose preference test

The sucrose preference test was performed as we described previously^22^ by exposing the mice to two bottles with either tap water or 1% sucrose, in a 2-h session at 2 and 24 h after cessation of vapor exposure. Each bottle was weighed before and after testing, and sucrose preference was calculated as the % sucrose consumed relative to the total liquid intake ((sucrose consumed (g)/total liquid consumed (g)) × 100) for each session.

### Operant conditioning using FED3

#### Equipment and setup

Operant conditioning was performed using the Feeding Experimentation Device 3 (FED3)^27–29^. The FED3 is a small battery-powered operant device that dispenses pellet rewards (*e.g.*, grain-based diet or sucrose) according to experimenter-designed programs. For this set of behaviors we used sucrose pellets (Dustless Precision Pellets, 20 mg Sucrose, Bio-Serv). The FED3 contains two nose pokes for operant training, a pellet well for reward retrieval, and light and sound cues upon reward delivery. All actions were timestamped and recorded to internal storage for future analysis. To habituate mice to the FED3 and increase the speed of acquisition of FR1 behavior^29^ mice were given 48 h of continuous access to a FED3 in their group-housed home cage. This device was set to the Free Feeding paradigm, where the removal of a pellet from the well triggers the release of a new pellet in the absence of any delivery cues. For all operant conditioning sessions (2-h sessions, starting 2 h after completion of vapor exposure) animals were individually placed into a standard cage containing a FED3 with iso-pad bedding under red light, then returned to group housing upon completion of the session. Experimental Parameters, such as FED3 error rate, were counterbalanced between treatment groups for each experiment. Animals which failed to reach acquisition by the end of the study were excluded from all analyses (AIR, n = 1; VEH, n = 1).

#### Fixed ratio 1 (FR1)

To establish operant responding for sucrose pellets (Dustless Precision Pellets, 20 mg Sucrose, Bio-Serv) mice were given daily access to the FED3 under a Fixed Ratio 1 (FR1) feeding paradigm, with a pellet in the well at the start of the session. The left nose poke was set as the active operandum and correct responses were paired with brief audio and visual cues serving as conditioned reinforcers. No timeout period after pellet nose poke or pellet retrieval was used. Acquisition criterion was defined as meeting or exceeding 75% accuracy in a rolling window of 20 pokes (e.g., 15/20 pokes correct). Animals which failed to reach acquisition were excluded (AIR, n = 1; VEH, n = 1). For each session, the number of pellets obtained, number of left pokes made, number of right pokes made, % correct, latency to first pellet retrieval, average pellet retrieval time, average inter-pellet interval, and number of sessions until meeting criterion were recorded.

#### Progressive ratio test (PRT)

To measure motivation for sucrose, the PRT was performed using an escalating reinforcement schedule for sucrose pellets. Progressive responding was measured over a single 4-h session occurring 2 h after completion of vaping exposure on Session 10. The number of responses on the active lever needed for reward dispensation increased exponentially based on the following equation: (ratio = ratio + round ((5 * exp (0.2 * PelletCount)—5))^29,30^. Breakpoint was defined as the highest number of reinforcers earned before a 2-h break between reinforcers. For this session, the total number of pellets obtained, number of left and right pokes made, percent correct, latency to first pellet retrieval, average pellet retrieval time, average inter-pellet interval, and breakpoint were recorded or calculated.

#### Quinine test (QT)

To evaluate the impact of aversive consequences on sucrose consumption, mice were given a single 2-h FR1 session using the FED3 dispensing sucrose pellets containing the bitterant quinine (Dustless Precision Pellets, 20 mg Sucrose 0.44% Quinine by weight, Bio-Serv) in place of the normal unadulterated sucrose pellets. The total number of pellets obtained, number of left and right pokes made, percent correct, latency to first pellet retrieval, average pellet retrieval time, average inter-pellet interval, and number of pellets eaten (calculated as the number of pellets taken from the device minus the number of pellets found on the floor of the cage at task completion) were recorded. A negative number of pellets eaten signifies that the mouse intercepted a pellet mid-air prior to it reaching the well, such that the FED3 could not record the pellets’ dispensation; the FED3 would then release another pellet and the mouse could retrieve two pellets for one active nose poke.

### Statistical analyses

Statistical analyses were performed using GraphPad Prism (version 10.1.0) and detailed reports are available in Supplemental Tables 1–3. All data are reported as mean ± SEM and individual data points are displayed where applicable. Behavioral experiments were analyzed as follows: for affective behaviors and tactile allodynia, one- or two-way ANOVA with repeated measures as appropriate and Tukey post hoc; for cognitive tests, Kruskal–Wallis followed by Dunn’s post hoc. Pharmacokinetic data are presented as follows: area under the curve, AUC (ng*hr/ml); maximum plasma concentration, Cmax (ng/ml); plasma half-life, t1/2 (min). The AUC and its relative statistical analysis were computed by the Center for Biostatistics and Health Data Science (CBHDS), Department of Statistics at Virginia Tech using the PK package in R, which is based on the work of Wolfsegger and Jaki^31^. Statistical outliers were determined using Grubbs’ Test. In the event of multiple outliers within the same treatment group, the individual with the higher z-value was removed. During the operant tasks nose pokes, pellets retrieved, and percent correct were considered linked, meaning one outlier per group per linked measure. Other measures were considered un-linked, and separately analyzed for outliers. The criteria for significance were as follows: *P < 0.05, **P < 0.01, ***P < 0.001, ****P < 0.0001.

## Results

### Optimization of the CVE paradigm

**Figure 1:**
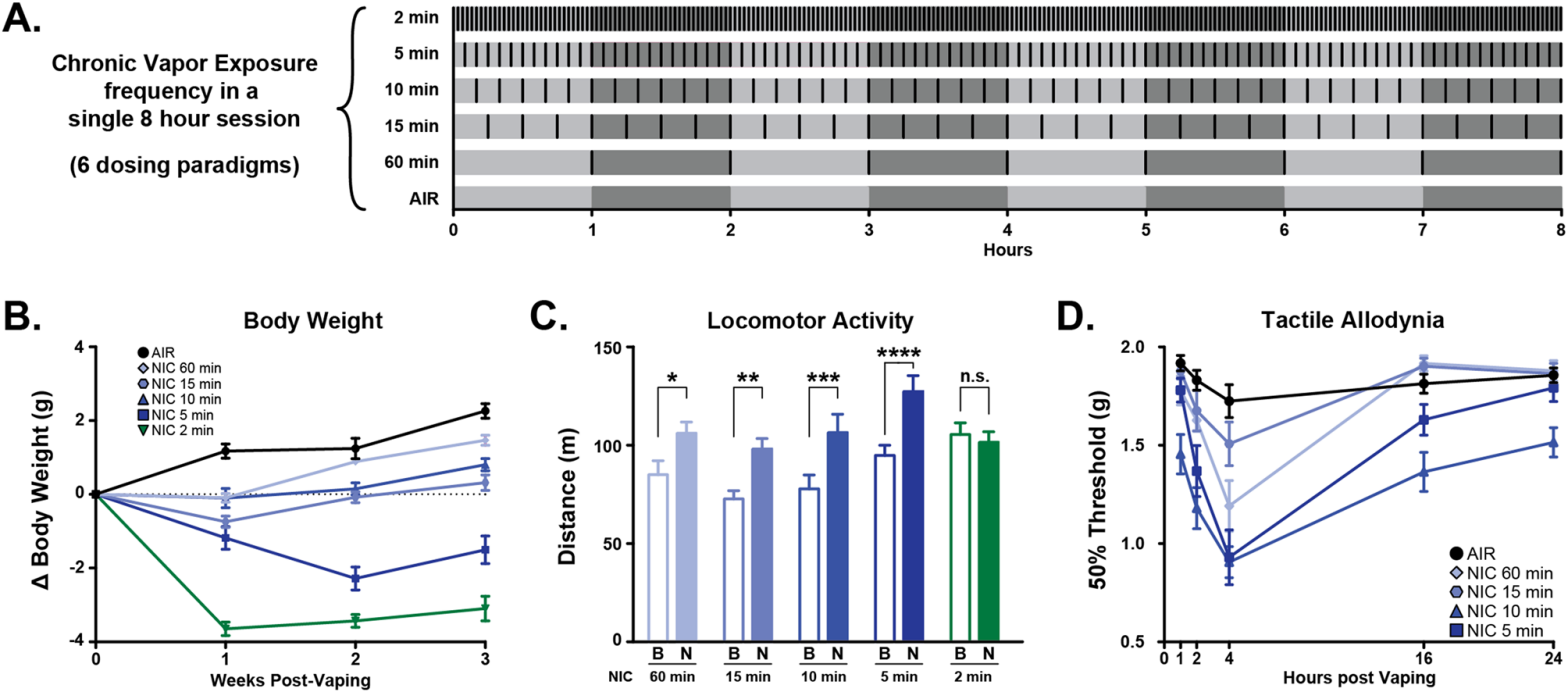
Dose-dependent effects of chronic vapor exposure (CVE) in mice. (**A**) Visualization of the chronic vapor exposure frequencies evaluated in this study during a single 8 h session with each nicotine (NIC or N) exposure indicated by a black line. The 6 dosing paradigms included air controls (AIR, 0 exposures), 60 min (8 exposures), 15 min (32 exposures), 10 min (48 exposures), 5 min (96 exposures), and 2 min (240 exposures). (**B**) Changes in body weight (g) during the first 3 weeks of CVE. (**C**) Locomotor activity prior to (baseline, B) and immediately after a single nicotine exposure session collected following a minimum of five weeks CVE. (**D**) Expression of tactile allodynia measured during post-exposure abstinence following a minimum of 6 weeks CVE. Data expressed as mean ± s.e.m., and statistical significance indicated by *p < 0.05, **p < 0.01, ***p < 0.001, ****p < 0.0001.

**Figure 2:**
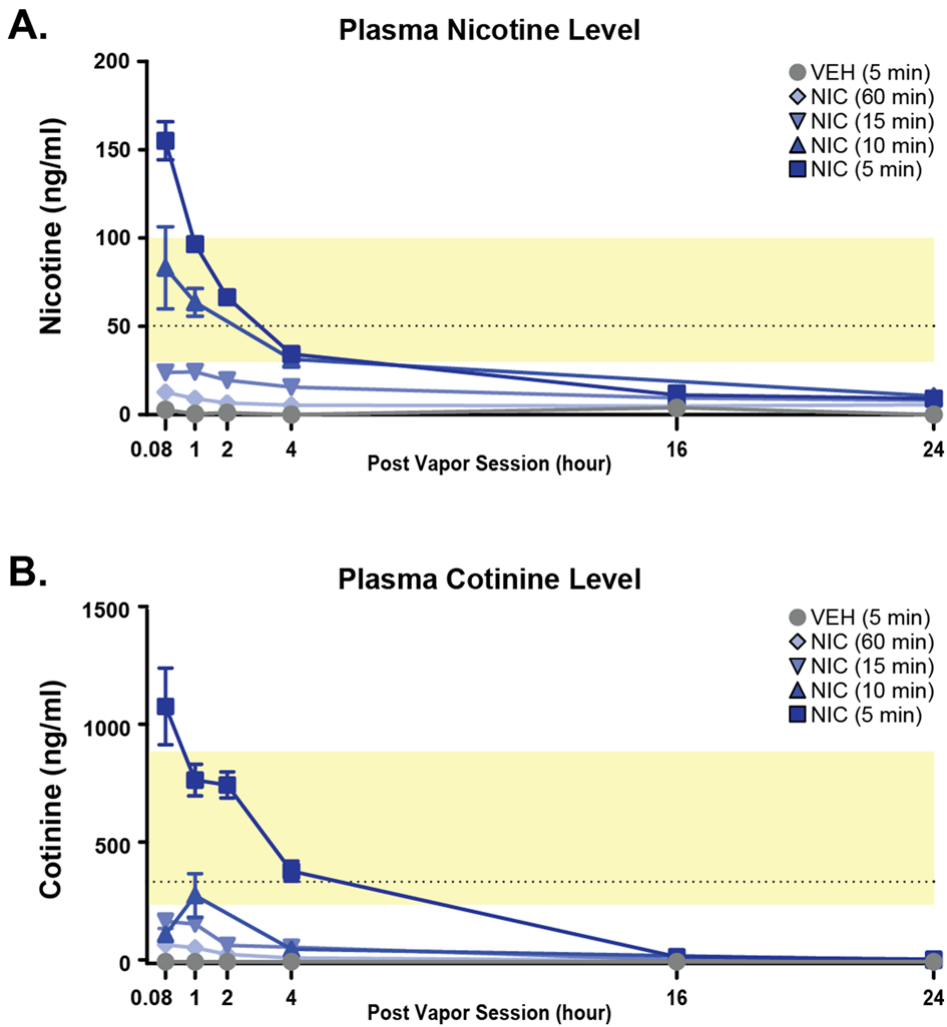
Pharmacokinetic analysis of plasma nicotine and cotinine following nicotine CVE. Mice exposed to different inter-vape intervals (5, 10, 15, and 60 min) were used to measure plasma levels of (**A**) nicotine (NIC) and (**B**) cotinine at 5 min and 1, 2, 4, 16, and 24 h after the last vapor exposure. The shaded yellow box indicates the expected range of plasma nicotine or cotinine for individual nicotine-dependent human users. Data expressed as mean ± s.e.m.

**Figure 3:**
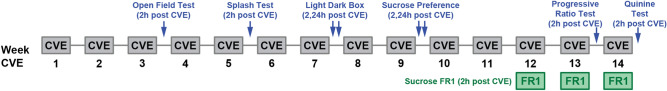
Timeline of the battery for affective and cognitive behavioral testing following CVE. Mice were exposed to nicotine (NIC) or vehicle (VEH) at a 10-min CVE inter-vape interval for 8 h daily sessions, 5 days per week, for 14 weeks. Additionally, a third group of mice were placed into chambers receiving room air (AIR) at the same time as other mice. Beginning at 3 weeks of CVE, mice were evaluated for anxiety-like behaviors (Open Field Test, Light–Dark Box), depression-like behaviors (Splash Test, Sucrose Preference Test), and cognitive behaviors (acquisition of Sucrose Fixed-Ratio 1 operant self-administration, Progressive Ratio Test, Quinine Test).

To determine optimal conditions for the CVE paradigm, we first examined the effects of 5 different nicotine vapor inter-vape intervals in different cohorts of mice (2–60 min, see Fig. 1A) on 3 validated output measures of nicotine effect: changes in body weight during each week of exposure, changes in locomotor activity, and the expression of tactile allodynia. Detailed statistical results are available in Supplemental Table 1. While all treatment groups receiving nicotine exhibited a significant body weight change compared to the air control, the 2-min frequency produced significant body weight loss (Fig. 1B) and did not increase locomotor activity relative to baseline (Fig. 1C), indicating that this dose was too high for further study. Accordingly, we proceeded to measure tactile thresholds to assess abstinence-induced hyperalgesia (Fig. 1D) and performed an in vivo pharmacokinetic study of blood levels of nicotine and its active metabolite cotinine at different time points following the last vapor exposure in all other inter-vape intervals (Fig. 2A,B, Table 1). While mice exposed to the 5-min nicotine frequency exhibited increased locomotor activity (Fig. 1C) and abstinence-induced tactile allodynia (Fig. 1D), they showed a significant reduction in body weight over time (Fig. 1B) and supraphysiological levels of nicotine and cotinine in the blood well above ranges found in human smokers (Fig. 2A,B, Table 1). Despite increased locomotor activity (Fig. 1C), mice subjected to 15-min or 60-min nicotine inter-vape intervals displayed body weight gain (Fig. 1B), did not develop significant allodynia (Fig. 1D) and failed to exhibit blood levels of nicotine and cotinine within range of human smokers (Fig. 2A,B). By contrast, mice given the 10-min nicotine frequency exhibited blunted weight gain, increased locomotor activity, and marked abstinence-induced tactile allodynia (Fig. 1B–D) along with comparable blood levels of nicotine and cotinine to human smokers (Fig. 2A,B, Table 1). Collectively, these results indicate that the delivery of vaporized nicotine at a 10-min frequency prevents body weight gain, elicits predicted increases in locomotor activity and abstinence-induced tactile allodynia, and generates nicotine blood levels equivalent to human smokers. Thus, we selected the 10-min inter-vape interval for evaluation of affective and cognitive behaviors using the nicotine CVE paradigm. A new cohort of mice were divided into three groups (n = 20 per group); the first group was exposed to CVE containing nicotine (NIC, 20 mg/ml), the second group was exposed to vehicle CVE without nicotine (VEH), and the third group was exposed only to passive airflow (AIR), all delivered at a 10-min inter-vape interval. An experimental timeline for these studies is presented in Fig. 3.

### Nicotine CVE does not elicit substantial changes in affective behaviors

**Figure 4:**
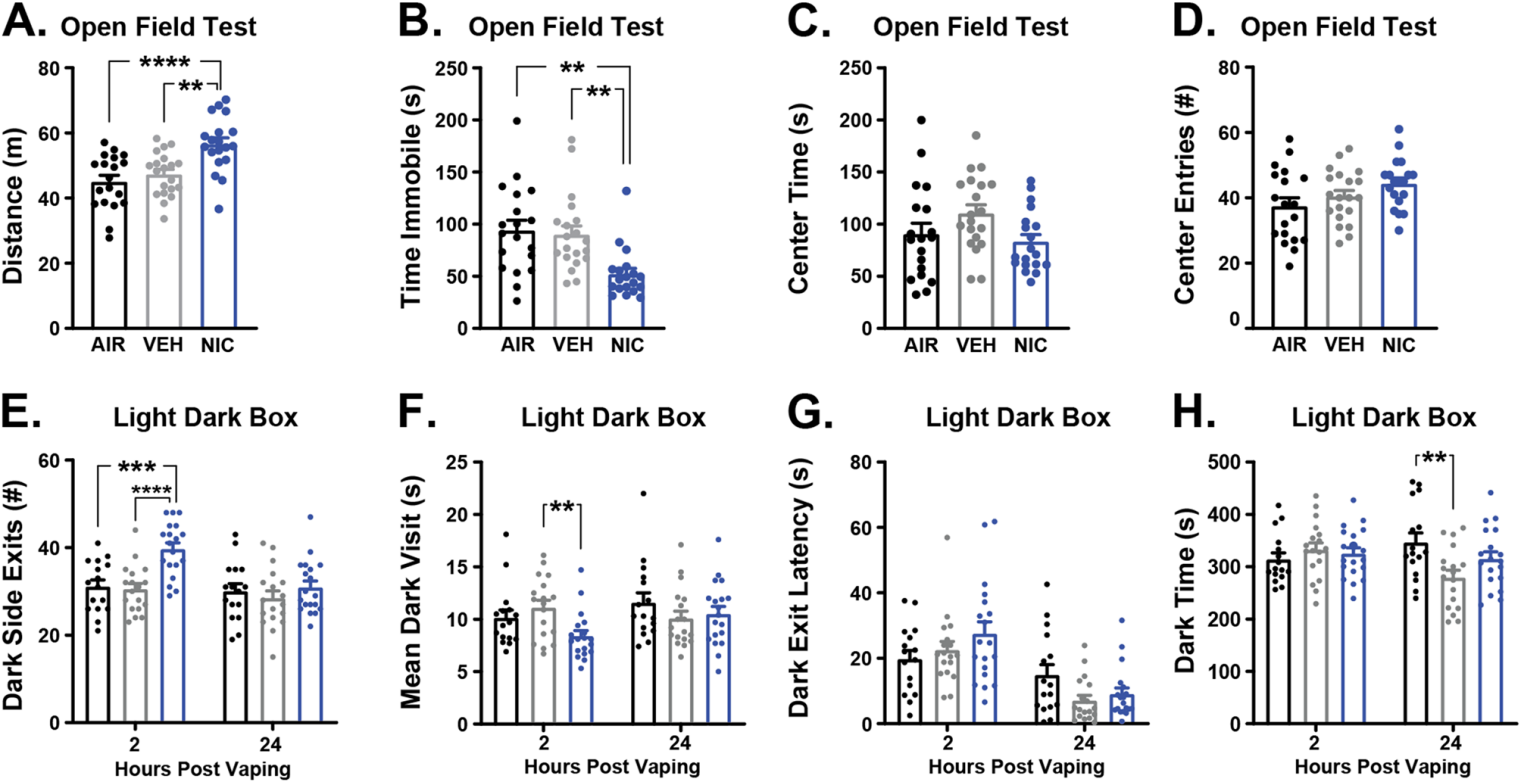
Effects of nicotine CVE on anxiety-like behaviors. (**A**–**D**) Open field test (OFT) at 2 h and (**E**–**H**) light–dark box test (LDT) at 2 h and 24 h after the last vapor exposure for nicotine CVE (NIC), vehicle CVE (VEH), and air controls (AIR). Output measures from the OFT include (**A**) distance traveled, (**B**) time spent immobile, (**C**) center time, and (**D**) number of center entries. Output measures from the LDT include (**E**) number of dark side exits, (**F**) the mean dark visit time, (**G**) dark exit latency, and (**H**) the total dark side time. Data expressed as mean ± s.e.m., and statistical significance indicated by **p < 0.01, ***p < 0.001, ****p < 0.0001. n = 18–20 per group.

Next, we investigated the effects of nicotine CVE on affective behaviors at defined timepoints after the last vapor exposure as follows: the Open Field Test (OFT) and the Splash Test at 2 h, the Light–Dark box Test (LDT) and Sucrose Preference Test (SPT) at 2 and 24 h. Detailed statistical results are available in Supplemental Table 2. In the OFT, distance traveled, and time spent immobile represent measures of activity, while time spent on the edges and the number of center entries signify levels of anxiety-like behavior. In the LDT, the number of exits from, average length of visits to, and latency to exit from the dark area were taken as measures of activity, while time spent in the dark portion signifies levels of anxiety-like behavior. As expected, there was no significant difference between vehicle CVE and air controls at 2 h after cessation of vapor exposure for any parameter examined in OFT (Fig. 4A–D) or LDT (Fig. 4E–H), indicating that the vehicle itself did not produce effects on anxiety-like behaviors or overall locomotor activity at this time point or inter-vape interval. Compared with vehicle CVE or air controls, mice receiving nicotine CVE displayed an increase in distance traveled and a decrease in immobility time (Fig. 4A–B), but there was no significant difference from controls in time spent in the center or number of center entries in the OFT (Fig. 4C,D). Similarly, in the LDT, nicotine CVE produced an increased number of exits from the dark and decreased the average length of dark visits (Fig. 4E,F) at 2 h after cessation of treatment, without altering the latency to the first dark exit or time spent in the dark at either 2 h or 24 h after cessation of treatment (Fig. 4G,H). Thus, this paradigm of nicotine CVE produced hyperactivity without effect on anxiety-like behaviors as evaluated by the OFT or LDT.

**Figure 5:**
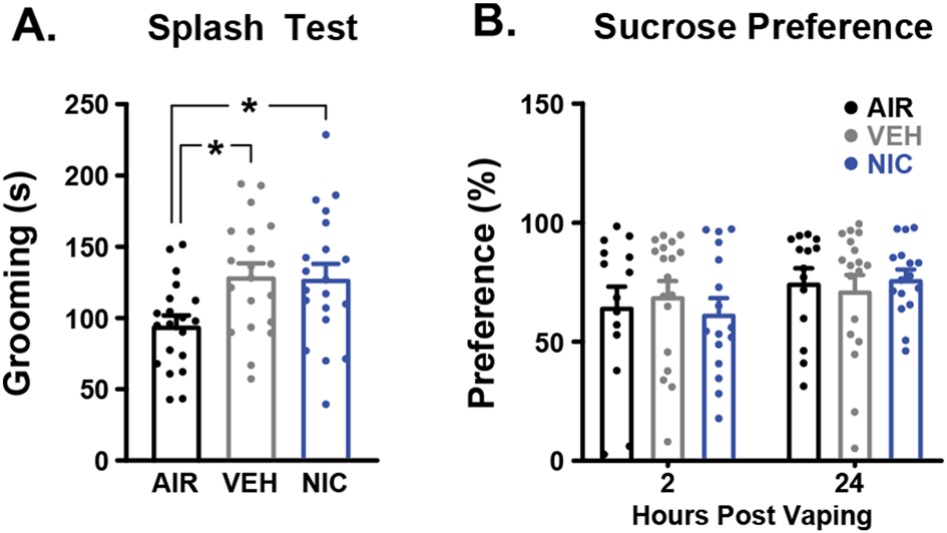
Effects of nicotine CVE on depression-like behaviors. Splash Test and Sucrose Preference Test were evaluated after the last vapor exposure for nicotine CVE (NIC), vehicle CVE (VEH), and air controls (AIR). (**A**) Total time spent grooming during the Splash test at 2 h post-CVE. (**B**) The preference for sucrose (as a percentage of total liquid consumption) during the sucrose preference test at 2 h or 24 h post-CVE. Data expressed as mean ± s.e.m., and statistical significance indicated by *p < 0.05. n = 14–18 per group.

To determine the effects of nicotine CVE on measures of anhedonia, the same groups of mice were subjected to the Splash Test 2 h, and Sucrose Preference Test at 2 and 24 h, after cessation of treatment. Detailed statistical results are available in Supplemental Table 2. In the Splash Test, both vehicle and nicotine spent significantly more time grooming than air controls (Fig. 5A). As there was no difference between vehicle and nicotine treatment groups, increased grooming is most likely due to fur and skin exposure to the vehicle itself. The vaporized vehicle solution is viscous, and is likely aversive to the mouse, possibly triggering a natural grooming response. There were no differences in sucrose preference between air and vehicle controls, and no significant effect of nicotine at 2 h or 24 h post vapor exposure (Fig. 5B), indicating that cessation from nicotine CVE did not produce anhedonia.

### Nicotine CVE impairs the acquisition of discrimination learning

**Figure 6:**
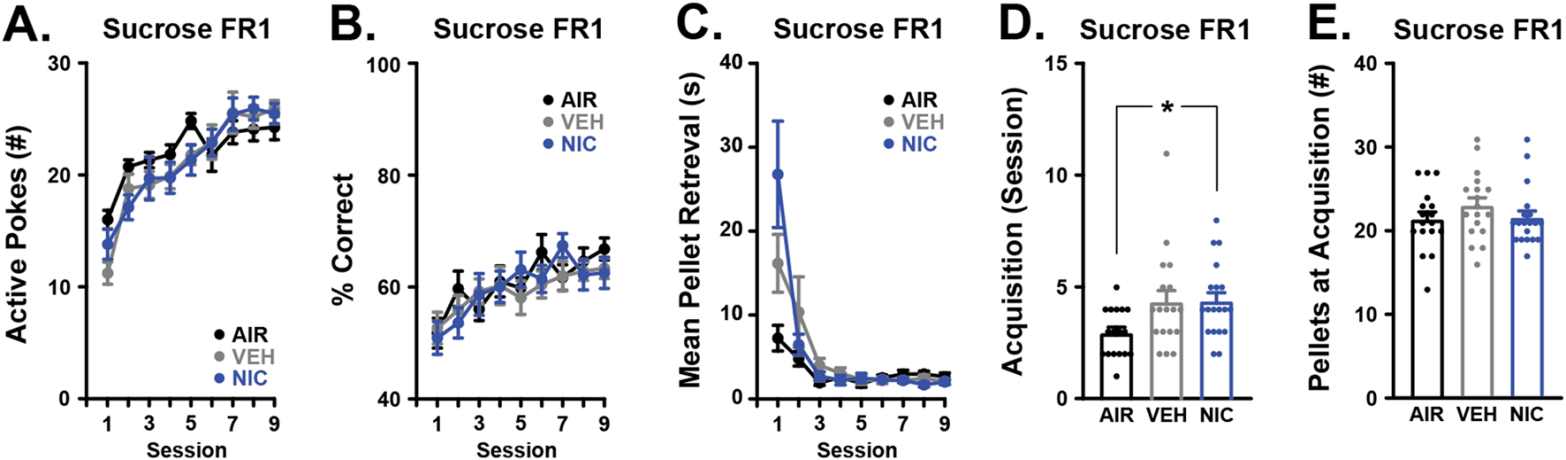
Effects of nicotine CVE on operant sucrose self-administration. Acquisition of operant self-administration of sucrose pellets was evaluated during daily 2-h sessions immediately following the last vapor exposure for nicotine CVE (NIC), vehicle CVE (VEH), or air controls (AIR). Output measures included (**A**) active nose pokes during each of the first 9 sessions, (**B**) response accuracy during each of the first 9 sessions, (**C**) mean time between the delivery and retrieval of the pellet during each of the first 9 sessions, (**D**) number of sessions until reaching the criteria for goal-directed responding (acquisition), and (**E**) the total number of pellets retrieved during the session that acquisition was achieved. Data expressed as mean ± s.e.m., and statistical significance indicated by *p < 0.05. n = 17–20 per group.

To examine the effects of nicotine CVE on operant learning, mice were trained to self-administer sucrose pellets using FED3 in daily 2-h sessions as previously described^29^. Detailed statistical results are available in Supplemental Tables 2 and 3. First, we evaluated the number of active nose pokes (Fig. 6A) and response accuracy (percentage of correct nose pokes) (Fig. 6B) in each training session to determine when mice achieved stable operant responding behavior, defined as meeting or exceeding 75% accuracy in a rolling window of 20 pokes. The first 9 sessions for animals are graphed to demonstrate the effects of training. With each successive training session, all groups exhibited faster pellet retrieval time after an active nose poke (Fig. 6C) with a significant effect of time but no main effect of treatment. Interestingly, there was a significant difference between nicotine CVE and air controls in the number of sessions until stable responding (Fig. 6D) with nicotine-treated mice needing more sessions to stability, although the vehicle may contribute in part to this effect. There were no effects of treatment on the number of pellets retrieved at stability (Fig. 6E), suggesting that the delayed acquisition observed in the nicotine group is not due to sucrose palatability. Analysis of the number of inactive nose pokes per session, number of pellets retrieved per session, latency to first pellet retrieval, and inter-pellet interval revealed significant effects of time, but only latency to first pellet retrieval showed significant effects of treatment (Fig. S1). These data suggest that nicotine CVE may impair the acquisition of operant behavior for sucrose rewards.

**Figure 7:**
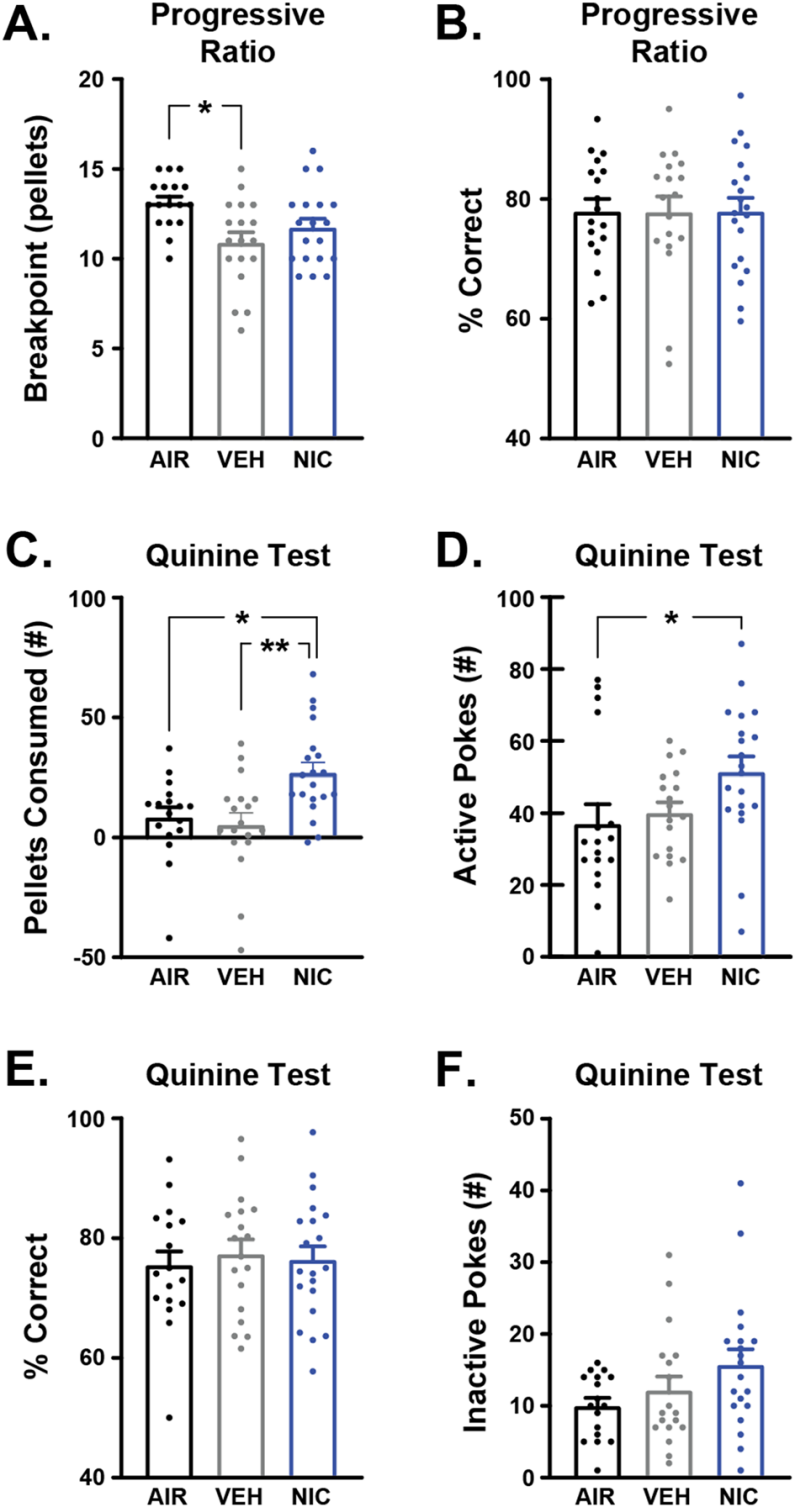
Effects of nicotine CVE abstinence on sucrose motivation. Motivation for sucrose was evaluated using the progressive ratio test (PRT) and the quinine test (QT), where sucrose pellets were adulterated with 0.44% quinine. Output measures for PRT included (**A**) breakpoint at the end of the session (4 h) and (**B**) Response accuracy expressed as the percentage of correct nose pokes. Response to altered taste preference during QT included (**C**) number of quinine-adulterated sucrose pellets consumed, (**D**) the number of active nose pokes, (**E**) response accuracy, and (**F**) inactive nose pokes. Data expressed as mean ± s.e.m., and statistical significance indicated by *p < 0.05, **p < 0.01. n = 17–20 per group.

After mice reached stable responding to FED3, they were subjected to a single 4-h session set to an escalating reinforcement schedule for 20 mg sucrose pellets (Progressive Ratio Test, PRT) to examine the effects of nicotine CVE on sucrose motivation as described previously^30^. Motivation for sucrose was measured by breakpoint, defined as the last pellet retrieved before a 2-h period without another pellet retrieval or the final number of pellets retrieved over the session. There was no effect of nicotine CVE on sucrose motivation (Fig. 7A) or ability to perform the PRT as measured by response accuracy/% correct (Fig. 7B). There were no significant differences between treatment groups in latency to first pellet retrieval, average pellet retrieval time, (Figure S2D-F) during the PRT, though differences in inter-pellet interval were observed between the air and nicotine groups. In contrast, we found a significant reduction in the number of active nose pokes made for vehicle CVE versus air controls (Figure S2A) resulting in fewer pellets retrieved at end of session (Figure S2C). Conversely, there was a corresponding increase in the number of inactive nose pokes made for air controls versus vehicle CVE (Figure S2B). Collectively, these data indicated that acute abstinence from nicotine CVE does not affect motivation for sucrose as a natural reward.

### Nicotine CVE increases consumption of quinine-infused pellets

To examine the effects of nicotine CVE on response to aversive consequences of sucrose consumption, mice were subjected to a single 2-h session of access to a FED3 on an FR1 reinforcement schedule with pellets consisting of 0.44% quinine in sucrose. Detailed statistical results are available in Supplemental Tables 2 and 3. There was a significant increase in consumption of quinine/sucrose pellets by nicotine CVE-treated versus both vehicle CVE-treated and air control mice (Fig. 7C). Additionally, nicotine CVE mice exhibited a greater number of active nose pokes than air controls (Fig. 7D). Despite an increased number of active nose pokes, there was no effect of treatment on response accuracy/% correct (Fig. 7E), number of inactive nose pokes made (Fig. 7F), average pellet retrieval time or the average inter-pellet interval (Figure S3B, C). However, vehicle CVE-treated mice demonstrated an increased latency to first pellet retrieval versus air and nicotine CVE-treated mice (Figure S3A). Additionally, while air and vehicle CVE mice demonstrated significant differences between sucrose and quinine/sucrose pellet consumption this effect was not observed in nicotine CVE-treated mice (Figure S3D). Taken together, these results indicate nicotine CVE alters quinine consumption patterns.

## Discussion

Nicotine and tobacco use are leading causes of preventable deaths, and a majority of those who attempt to quit using nicotine or tobacco will relapse. Electronic cigarettes and other electronic nicotine delivery systems (ENDS) were presented as a safer alternative to smoking cigarettes but have instead contributed to increasing nicotine use among US youths. However, these devices represent useful tools for creating preclinical methods of chronic nicotine exposure that better model human nicotine consumption. Using an ENDS device, we demonstrate that chronic, intermittent vapor-based nicotine exposure at a 10-min inter-vape interval produces expected changes in weight, locomotor activity, and plasma nicotine levels, as well as tactile allodynia following vaping cessation. While this dosing schedule results in deficits in operant acquisition and quinine perception, it does not produce changes in affective behavior.

Our CVE procedures produced dose-dependent pharmacokinetic and behavioral responses to nicotine. By manipulating the frequency of vapor exposure during a session (using a constant flow rate, vape time, and concentration of nicotine in the ENDS solution), we can produce dose-dependent changes in the Cmax and AUC. At exposure frequencies of 5 and 10 min, male mice metabolize nicotine at a similar rate as previously published work using other routes of administration^9^. Interestingly, we discovered that the half-life was much longer at a lower exposure frequency, which suggests that in-cage CVE may exhibit non-linear pharmacokinetics in mice. This could be attributed in part to oral consumption or absorption through the skin, a possibility that cannot be excluded using this inhalation model. At high-frequency dosing, we see a non-linear impact on nicotine metabolism as cotinine levels rise well beyond those typically seen in rodent or clinical studies at 1200 ng/ml. Collectively, our pharmacokinetic studies indicate that the 10-min CVE dosing frequency produces plasma nicotine levels at levels consistent with nicotine dependence in human and rodent models^9,32^.

Our systematic evaluation of multiple dosing regimens using physiological and behavioral output measures combined with pharmacokinetic analyses support chronic dosing at a 10-min frequency to produce relevant phenotypic changes associated with nicotine CVE in mice. We demonstrated that CVE to nicotine produces dose-dependent effects on body weight during the first three weeks of exposure, recapitulating a long-standing clinical observation^33,34^. These results are congruent with several reports that nicotine drives anorexic effects^35,36^ as well as consumption-independent effects on metabolism and activity^37,38^. The emergence of pain-like behavior during abstinence has been established as a key indicator of dependence, and our data show a clear dose-dependent increase in tactile allodynia following CVE with a peak at 2–4 h post-session. This supports multiple prior studies showing that nicotine abstinence or withdrawal produces increased allodynia in humans^39,40^ and rodents^41–43^. In contrast, we did not observe dose-dependent effects on locomotor activity, as all frequencies of nicotine exposure (except for the 2-min inter-vape interval) produced comparable increases in locomotor activity. Multiple studies report that chronic nicotine exposure can result in locomotor sensitization^44–46^. Elevated locomotor activity may be attributable to increased central and peripheral monoaminergic signaling, as acute nicotine exposure can elevate noradrenergic signaling in the periphery as well as facilitate dopamine release into the nucleus accumbens to drive drug reinforcement^47–49^. The minimum cumulative dose needed for dependence far exceeds the level for nicotine reinforcement behavior, suggesting that locomotor activity may serve as a poor criterion for dependence dose selection. Likewise, CVE at the 2-min exposure frequency produced nicotine plasma levels well in excess of those typically measured in humans and rodent models, and the observed decreased locomotor activity may reflect acutely depressed respiratory function seen in other high-dose nicotine exposure paradigms^50,51^. Thus, the CVE procedures and time points selected for evaluating the effect of nicotine inhalation exposure in this study were supported by multiple pharmacokinetic and behavioral output measures.

Our study revealed that nicotine CVE at a 10 min inter-vape interval did not result in nicotine-specific changes in affective behavior as measured by an Open Field Test, Light/Dark box Test, Sucrose Preference Test, or Splash test. However, we did observe vapor-specific effects during the splash test, whereby both vehicle and nicotine CVE groups demonstrated increased self-grooming at 2 h of abstinence. While increased self-grooming during splash/spray tests can be interpreted as increased motivation and self-care behavior^52^ or a reduction in depression-like behaviors^53^, the absence of reduced depression-like behavior during the sucrose preference test indicates that the observed results might better reflect self-grooming after completion of the vaping session performed to remove the viscous vehicle from the fur. Upon cessation of nicotine intake humans experience a number of side effects (*e.g.*, headache, increased anxiety and depression, cognitive deficits) which can serve as powerful negative reinforcers, contributing to continued nicotine use^46,54,55^.

While traditional models of chronic nicotine exposure generally report increased anxiety- and depression-like behaviors in response to nicotine cessation or abstinence^56,57^, chronic vapor-based exposure models demonstrate mixed findings. Some studies report that cessation results in increased anxiety-like behavior as measured by a novelty-suppressed feeding^20^ or by the elevated plus maze^58,59^. However, others have reported findings similar to ours, whereby vapor-based nicotine exposure resulted in body-weight changes and physical signs of withdrawal but did not result in significant differences between groups during the open-field test^43^. Such differences in findings may be due to any one of several factors. For instance, while the field of nicotine research has reached a general agreement on accepted dosing and duration parameters for subcutaneously implanted osmotic pumps or oral administration, these parameters have not been established for vapor exposure. Differences between labs regarding nicotine content, puff length, inter-vape interval, chamber airflow, and session length may alter nicotine pharmacokinetics^60^, confounding the direct comparison of results. Direct comparisons may be further confounded by measuring behavioral effects at different time points following treatment. For instance, we examined affective behaviors at 2- or 24-h post-vapor cessation, while others have conducted these studies immediately following the final vapor exposure^20^. Therefore, our observations of the lack of nicotine-specific changes in affective behaviors may reflect aspects of the study design in addition to the overall effects of vapor-based nicotine exposure.

However, these mixed observations may reflect a trend observed in human studies which show that electronic cigarette users report fewer withdrawal symptoms^61^, fewer cravings^62^ and less difficulty refraining from use^63^ than traditional cigarette users. Additionally, former smokers report that electronic cigarettes produce lower dependence^64^. Thus, it has been suggested that nicotine CVE may produce a dependence and motivation profile which is less driven by negative reinforcement than traditional cigarettes. This is not to say that electronic cigarettes are free from risk, as users still report signs of dependence and withdrawal. Future investigations are required to identify a rodent exposure paradigm that precisely captures this phenomenon.

Importantly, our study revealed that nicotine CVE impaired the acquisition of operant discrimination learning for sucrose. There were no sustained impacts of treatment on responding on the inactive nose poke, a proxy for general hyperactivity^65^, suggesting that operant behavior was not driven by hyperactivity. However, we cannot exclude this possibility, as mice demonstrated increased locomotor activity in the OFT at a similar time point. Additionally, the mice exhibited a similarly high preference for sucrose solution in the sucrose preference test, they consumed the same number of sucrose pellets (following the acquisition of discrimination learning), and they exhibited similar motivation for sucrose pellets during the progressive ratio test. Thus, delayed acquisition of discrimination learning most likely results from nicotine-induced cognitive impairments. Downregulation of β2-containing nicotinic acetylcholine receptors following CVE may participate in this effect, as deletion of the β2-subunit slows the acquisition of auditory discrimination learning for saccharin in mice^66^. Using T-Maze, a similar deletion of the β2-subunit in the dorsal striatum slows the acquisition of discrimination learning suggesting a potential role for this brain site^67^. While these studies showed no impact on cognitive flexibility using reversal learning tests, others have found that chronic high-dose nicotine exposure via minipump can impair cognitive flexibility in rats^68^. Future studies may evaluate CVE-induced impairments of these or other aspects of executive function.

While nicotine CVE did not affect the palatability of sucrose, nicotine exposure did impact the devaluation of sucrose rewards by quinine. Previous studies have shown that nicotine and quinine both produce a bitter taste by activating gustatory TRPM5 receptors^69,70^. Consistent with these findings, we show that nicotine-exposed mice exhibit increased consumption and active responding for sucrose pellets containing quinine compared to control groups, suggesting that nicotine CVE alters bitterness perception. As nicotine and quinine may have a similar bitter taste profile, the nicotine group may have consumed more quinine pellets due to an association between bitter taste and nicotine. However, these findings may be influenced by both sex^71^ and strain^72^ and thus warrant further investigation for a more complete understanding of this phenomenon. Other explanations for increased quinine consumption after nicotine CVE may include nicotine-induced impulsivity or behavioral disinhibition, as has been observed in rodent models of acute^73^ and chronic^68,74^ nicotine exposure, or cessation-induced changes in compulsive-like behaviors^75^ resulting in continued pellet retrieval and eating.

There are some limitations of the present work that can be addressed in future studies. While our current approach of modulating the frequency of exposure to increase the cumulative dose produced pharmacologically relevant plasma levels during exposure and increased tactile allodynia during abstinence, future studies may focus on modulating other parameters to facilitate more robust negative affective responses during abstinence. Technical aspects such as flow rate, exposure time, and chamber size may impact the nicotine pharmacokinetics^60^, necessitating further characterization of vapor exposure models. While our vapor exposure model did not produce robust signs of anxiety- and depression-like behavior, it still presents a useful tool for investigating the effects of CVE on cognitive impairments which may not be performed as easily using alternative models that produce only a single withdrawal period. We only evaluated a limited number of aspects of executive function, and future work can expand on these findings to determine the effect of CVE on cognitive flexibility, reward valuation and devaluation, attention, and response inhibition. Finally, these studies were only performed in male mice and comparative studies in female mice warrant further investigation.

### Supplementary Information

Supplementary Information.

## Data Availability

Data will be made available upon request.
