# Supplementary material for: Effect of chronic vapor nicotine exposure on affective and cognitive behavior in male mice
Source: Sci Rep. 2024 Mar 19;14:6646. doi: 10.1038/s41598-024-56766-z (PMC10951409; doi:10.1038/s41598-024-56766-z)
Supplement: Supplementary file 1 — Supplementary Information. [file 41598_2024_56766_MOESM1_ESM.docx]

**SUPPLEMENTAL INFORMATION**

**Effect of chronic vapor nicotine exposure on affective and cognitive behavior in male mice**

Laura B. Murdaugh^1,2,5^, Cristina Miliano^1,5^, Irene Chen^1^, Christine L. Faunce^1^, Luis A. Natividad^4^, Ann M. Gregus^1^*, and Matthew W. Buczynski^1,3^*

Affiliations:

^1^School of Neuroscience, Virginia Polytechnic Institute and State University, Blacksburg, Virginia, USA

^2^Translational Biology, Medicine, and Health, Virginia Polytechnic Institute and State University, Blacksburg, Virginia, USA

^3^Department of Chemistry, Virginia Polytechnic Institute and State University, Blacksburg, Virginia, USA

^4^College of Pharmacy, Division of Pharmacology and Toxicology, University of Texas at Austin, Austin, Texas, USA

^5^These authors contributed equally: Laura B. Murdaugh and Cristina Miliano.

*To whom correspondence should be addressed:

Matthew W. Buczynski: mwb@vt.edu

Ann M. Gregus: agregus@vt.edu

970 Washington St SW, Life Sciences I, Blacksburg, VA 24061 USA

Phone: (540) 231-2223

Fax: (540) 231-1475

Number of supplemental figures: 3; Number of supplemental tables: 3

Keywords: ENDS, e-cigarette, nicotine, vaping, cognitive, addiction


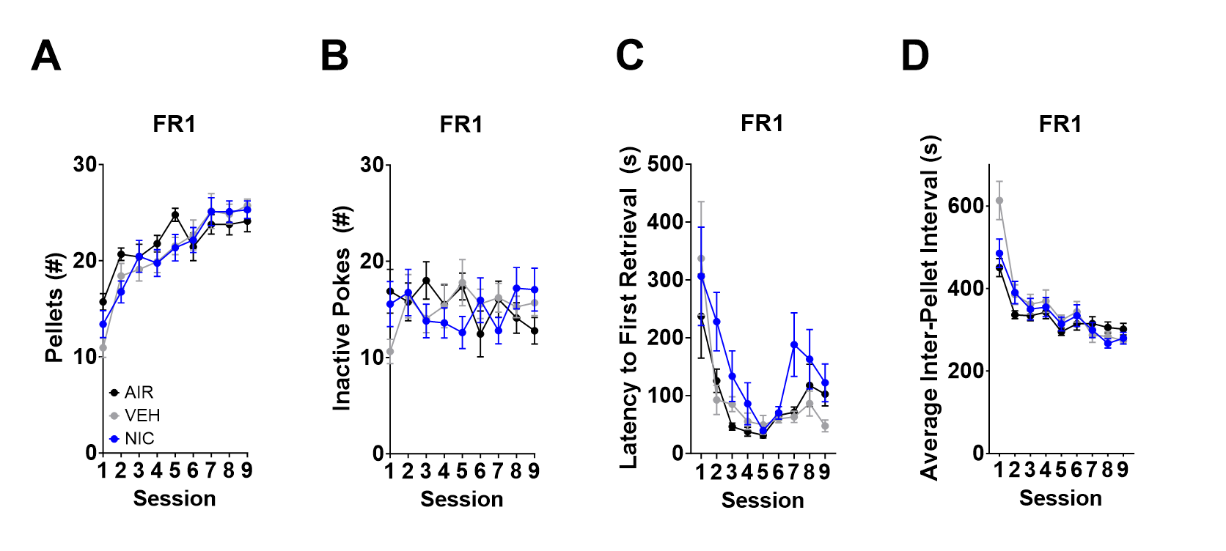


**Figure S1: Effects of nicotine CVE abstinence on measures of operant sucrose self-administration.** Acquisition of operant self-administration of sucrose pellets was evaluated during daily 2-hour sessions immediately following the last vapor exposure for nicotine CVE (NIC), vehicle CVE (VEH), or air controls (AIR). Output measures included **(A)** total sucrose pellets retrieved during each of the first 9 sessions, **(B)** number of inactive pokes during each of the first 9 sessions, **(C)** latency to remove the already-present sucrose pellet from the well during each of the first 9 sessions, and **(D)** average time between pellet dispensations during each of the first 9 sessions. Data expressed as mean ± s.e.m. n=12-20 per group per session.


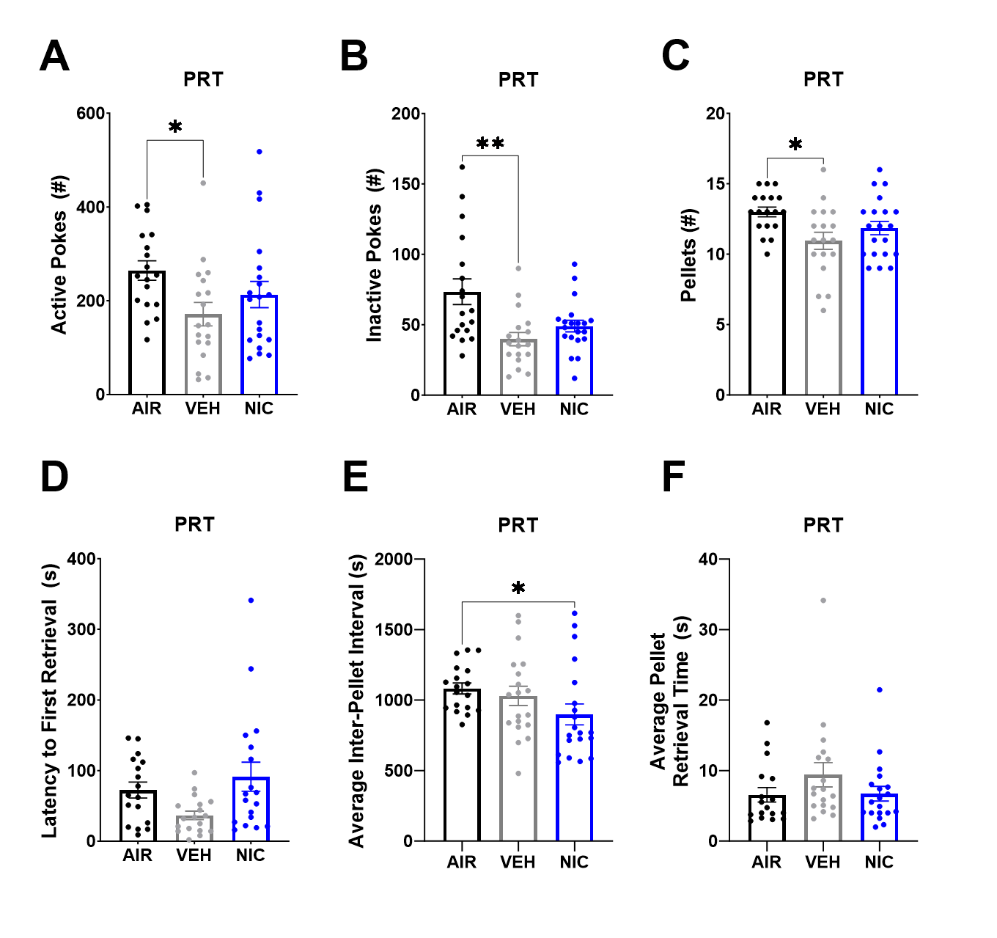


**Figure S2: Effects of nicotine CVE abstinence on measures during a test of sucrose motivation.** Motivation for sucrose was evaluated using a Progressive Ratio Test (PRT). Output measures for the PRT include **(A)** active nose pokes during the PRT, **(B)** inactive nose pokes during the PRT, **(C)** total sucrose pellets retrieved during the PRT, **(D)** latency to remove the already-present sucrose pellet from the well during the PRT, **(E)** average time between pellet dispensations during the PRT, and **(F)** mean time between the delivery and retrieval of the pellet during the PRT. Data expressed as mean ± s.e.m., and statistical significance indicated by * p<0.05, ** p<0.01. n = 17-20 per group.


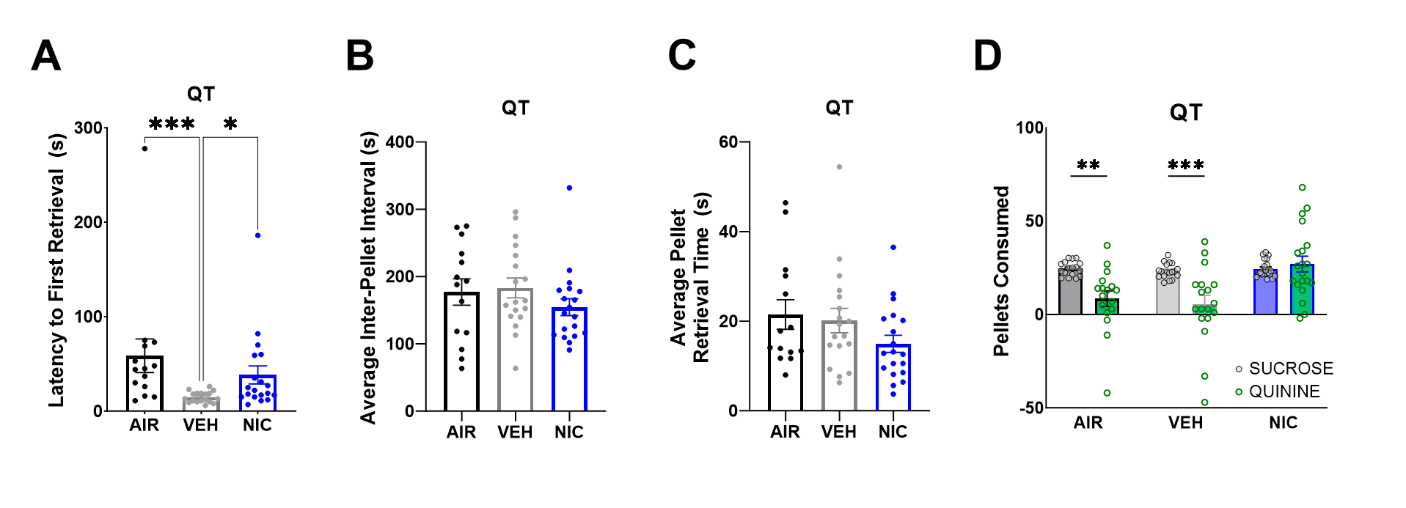


**Figure S3: Effects of nicotine CVE abstinence on measures during a test of sucrose motivation and response to aversive reward.** Motivation for sucrose and response to reward devaluation was evaluated using the quinine test (QT), where sucrose pellets were adulterated with 0.44% quinine. Output measures for the QT included **(A)** latency to remove the already present quinine-adulterated sucrose pellet during the QT, **(B)** average time between pellet dispensations during the QT, **(C)** mean time between the delivery and retrieval of the pellet during the QT, and **(D)** comparison of pellet consumption from prior sucrose days (filled, grey) and QT (open, green) between groups. Data expressed as mean ± s.e.m., and statistical significance indicated by * p<0.05, ** p<0.01, *** p<0.005. n =14-20 per group.

**Supplemental Table 1 – Statistics for dose-dependent effects of CVE in mice (Fig. 1)**

| **Fig.** | **Task** | **Statistical Test** | **Factor** | **F-Value** | **P-Value** | **Post-Hoc**  **(Displayed if P<0.1)** |
| --- | --- | --- | --- | --- | --- | --- |
| **1B** | Body Weight change (g) by Week of Vaping Dose Curve | Two-Way RM ANOVA | Interaction | F (15, 447) = 24.07 | <0.0001 | Dunnet’s Multiple Comparisons test  Week 1  AIR1 vs. NIC (2 min): P=<0.0001 ^^^^  AIR vs. NIC (5 min): P=<0.0001 ####  AIR vs. NIC (10 min): P=<0.0001 ****  AIR vs. NIC (15 min): P=<0.0001 + + + +  AIR vs. NIC (60 min): P=<0.0001 $$$$  Week 2  AIR vs. NIC (2 min): P=<0.0001 ^^^^  AIR vs. NIC (5 min): P=<0.0001 ####  AIR vs. NIC (10 min): P=.0004 ***  AIR vs. NIC (15 min): P=<0.0001 + + + +  Week 3  AIR vs. NIC (2 min): P=<0.0001 ^^^^  AIR vs. NIC (5 min): P=<0.0001 ####  AIR vs. NIC (10 min): P=<0.0001 ****  AIR vs. NIC (15 min): P=<0.0001 + + + +  AIR vs. NIC (60 min): P=0.0173 $ |
|  |  |  | Weeks of CVE | F (3, 447) = 26.83 | <0.0001 |  |
|  |  |  | CVE Dose | F (5, 447) = 193.5 | <0.0001 |  |
| **1C** | Locomotor Activity (m) Dose Curve | Two-Way RM ANOVA | Pre-Post x Dose | F (4, 90) = 4.480 | 0.0024 | Bonferroni’s Multiple Comparisons test  BL – POST  NIC (5 min): P<0.0001 ****  NIC (10 min): P=0.0007 ***  NIC (15 min): P=0.0031 **  NIC (60 min): P=0.0163 * |
|  |  |  | Pre-Post | F (1, 90) = 44.05 | <0.0001 |  |
|  |  |  | CVE Dose | F (4, 90) = 3.968 | 0.0052 |  |
|  |  |  | Subject | F (90, 90) = 2.090 | 0.0003 |  |
| **1D** | 50% Response Threshold (g) Dose Curve | Two-Way RM ANOVA | Hours Post CVE x CVE Dose Factor | F (16, 340) = 2.885 | 0.0002 | Dunnet’s Multiple Comparisons test  1 hour  AIR vs. NIC (10 min): P=0.0008 ***  2 hour  AIR vs. NIC (5 min): P=0.0102 #  AIR vs. NIC (10 min): P=<0.0001****  AIR vs. NIC (60 min): P=0.0372 $  4 hour  AIR vs. NIC (5 min): P=0.0001 ###  AIR vs. NIC (10 min): P=<0.0001****  AIR vs. NIC (60 min): P=0.0063 $$  16 hour  AIR vs. NIC (10 min): P=0.0014**  24 hour  AIR vs. NIC (10 min): P=0.0013** |
|  |  |  | Hours Post CVE | F (3.211, 273.0) = 46.39 | <0.0001 |  |
|  |  |  | CVE Dose | F (4, 85) = 18.57 | <0.0001 |  |
|  |  |  | Subject | F (85, 340) = 2.549 | <0.0001 |  |

**Supplemental Table 2 – Statistics for the Main figures (Figs. 4-7).**

| **Fig.** | **Task** | **Statistical Test** | **Factor** | | **F-Value** | | **P-Value** | | **Post-Hoc**  **(Displayed if P<0.1)** |
| --- | --- | --- | --- | --- | --- | --- | --- | --- | --- |
| **4A** | **OFT**  Distance Traveled (m) | One-Way ANOVA | All Groups | | F (2, 55) = 11.63 | | <0.0001 | | Tukey’s Multiple Comparisons test  AIR vs. NIC: P<0.0001 ****  VEH vs. NIC: P=0.0014 ** |
| **4B** | **OFT**  Time Immobile (s) | One-Way ANOVA | All Groups | | F (2, 55) = 8.334 | | 0.0007 | | Tukey’s Multiple Comparisons test  AIR vs. NIC: P=0.0015 **  VEH vs. NIC: P=0.0038 ** |
| **4C** | **OFT**  Time in Center (s) | One-way ANOVA | All Groups | | F (2, 55) = 2.732 | | 0.0739 | | Tukey’s Multiple Comparisons test  VEH vs. NIC: P=0.0717 |
| **4D** | **OFT**  Center Entries (#) | One-Way ANOVA | All Groups | | F (2, 55) = 2.757 | | 0.0723 | | Tukey’s Multiple Comparisons test  AIR vs. NIC: P=0.0585 |
| **4E** | **LDT**  Exits from Dark (#) | Two-Way RM ANOVA | Hours Post CVE x Treatment | | F (2, 50) = 5.221 | | 0.0087 | | Tukey’s Multiple Comparisons test  2 hour  AIR vs. NIC: P=0.0003 ***  VEH vs. NIC: P<0.0001 **** |
|  |  |  | Hours Post CVE | | F (1, 50) = 13.40 | | 0.0006 | |  |
|  |  |  | Treatment | | F (2, 50) = 7.576 | | 0.0013 | |  |
|  |  |  | Subject | | F (50, 50) = 1.530 | | 0.0681 | |  |
| **4F** | **LDT**  Average Dark Visit (s) | Two-Way RM ANOVA | Hours Post CVE x Treatment | | F (2, 50) = 4.900 | | 0.0114 | | Tukey’s Multiple Comparisons test  2 hour  VEH vs. NIC: P=0.0171 * |
|  |  |  | Hours Post CVE | | F (1, 50) = 3.727 | | 0.0592 | |  |
|  |  |  | Treatment | | F (2, 50) = 1.627 | | 0.2068 | |  |
|  |  |  | Subject | | F (50, 50) = 2.486 | | 0.0008 | |  |
| **4G** | **LDT**  Latency to Dark Exit (s) | Two-Way RM ANOVA | Hours Post CVE x Treatment | | F (2, 50) = 5.818 | | 0.0054 | |  |
|  |  |  | Hours Post CVE | | F (1, 50) = 59.52 | | <0.0001 | |  |
|  |  |  | Treatment | | F (2, 50) = 0.6739 | | 0.5143 | |  |
|  |  |  | Subject | | F (50, 50) = 2.359 | | 0.0015 | |  |
| **4H** | **LDT**  Time in Dark (s) | Two-Way RM ANOVA | Hours Post CVE x Treatment | | F (2, 50) = 8.163 | | 0.0009 | | Tukey’s Multiple Comparisons test  24 hour  AIR vs. VEH: P=0.0026 ** |
|  |  |  | Hours Post CVE | | F (1, 50) = 1.484 | | 0.2289 | |  |
|  |  |  | Treatment | | F (2, 50) = 1.104 | | 0.3395 | |  |
|  |  |  | Subject | | F (50, 50) = 2.453 | | 0.0009 | |  |
| **5A** | **Splash Test**  Grooming (s) | One-Way ANOVA |  | | F (2, 57) = 4.908 | | 0.0108 | | Tukey’s Multiple Comparisons test  AIR vs. VEH: P=0.0197 *  AIR vs. NIC: P=0.0284 * |
| **5B** | **Sucrose Preference**  Sucrose Preference (%) | Two-Way RM ANOVA | Hours Post CVE x Treatment | | F (2, 45) = 0.7104 | | 0.4969 | |  |
|  |  |  | Hours Post CVE | | F (1, 45) = 4.267 | | 0.0446 | |  |
|  |  |  | Treatment | | F (2, 45) = 0.01634 | | 0.9838 | |  |
|  |  |  | Subject | | F (45, 45) = 1.761 | | 0.0304 | |  |
| **6A** | **FR1**  Active Pokes (#) Per Day by Group | Two-Way RM ANOVA | Session × Treatment | F (16, 432) = 2.101 | | 0.0076 | | Tukey’s Multiple Comparisons test  Session 1  AIR vs. VEH: P=0.0151 *  Session 2  AIR vs. NIC: P=0.0836 | |
|  |  |  | Session | F (8, 432) = 45.02 | | <0.0001 | |  |  |
|  |  |  | Treatment | F (2, 54) = 0. 4028 | | 0.6704 | |  |  |
|  |  |  | Subject | F (54, 432) = 5.774 | | <0.0001 | |  |  |
| **6B** | **FR1**  Percent Correct (%) Per Day by Group | Two-Way RM ANOVA | Session × Treatment | F (16, 432) = 0.8673 | | 0.6078 | |  | |
|  |  |  | Session | F (8, 432) = 8.339 | | <0.0001 | |  |  |
|  |  |  | Treatment | F (2, 54) = 0.2863 | | 0.7521 | |  |  |
|  |  |  | Subject | F (54, 432) = 2.669 | | <0.0001 | |  |  |
| **6C** | **FR1**  Average Pellet Retrieval Time (s) Per Day by Group | Mixed Effects Model (REML) | Session × Treatment | F (16, 426) = 4.257 | | <0.0001 | | Tukey’s Multiple Comparisons test  Session 1  AIR vs. VEH: P=0.0658  AIR vs. NIC: P=0.0188 *  Session 3  AIR vs. VEH: P=0.0673  Session 8:  AIR vs. NIC: P=0.0170 * | |
|  |  |  | Session | F (1.727, 91.98) = 25.27 | | <0.0001 | |  |  |
|  |  |  | Treatment | F (2, 54) = 2.473 | | 0.0938 | |  |  |
| **6D** | **FR1**  Sessions until Stable (#) | Kruskal-Wallis Test | All Groups | Kruskal-Wallis Statistic  H(2) = 7.778 | | 0.0205 | | Dunn’s Multiple Comparisons test  AIR vs. NIC: P=0.0262 *  AIR vs. VEH: P=0.0886 | |
| **6E** | **FR1**  Pellets Retrieved at Stability (#) | One-Way ANOVA | All Groups | F (2, 54) = 1.951 | | 0.3996 | |  | |
| **7**  **A** | **PRT**  Breakpoint (2 hours, Pellets, #) | Kruskal-Wallis Test | All Groups | Kruskal-Wallis Statistic  H(2) = 9.029 | | 0.0109 | | Dunn’s Multiple Comparisons test  AIR vs. VEH: P=0.0114 *  AIR vs. NIC: P=0.0842 | |
| **7B** | **PRT**  Percent Correct (%) | Kruskal-Wallis Test | All Groups | Kruskal-Wallis Statistic  H(2) = 0.03749 | | 0.9814 | |  | |
| **7C** | **QT**  Pellets Consumed  (# Retrieved - # On Floor) | Kruskal-Wallis Test | All Groups | Kruskal-Wallis Statistic  H(2) = 12.43 | | 0.0020 | | Dunn’s Multiple Comparisons test  AIR vs. NIC: P=0.0172 *  VEH vs. NIC: P=0.0038 ** | |
| **7D** | **QT**  Active Pokes (#) | Kruskal-Wallis Test | All Groups | Kruskal-Wallis Statistic  H(2) = 7.664 | | 0.0217 | | Dunn’s Multiple Comparisons test  AIR vs. NIC: P=0.0232 * | |
| **7E** | **QT**  Percent Correct (%) | Kruskal-Wallis Test | All Groups | Kruskal-Wallis Statistic  H(2) = 0.3456 | | 0.8413 | |  | |
| **7F** | **QT**  Inactive Pokes (#) | Kruskal-Wallis Test | All Groups | Kruskal-Wallis Statistic  H(2) = 4.760 | | 0.0925 | |  | |

**Supplemental Table 3 – Statistics for the Supplemental figures (Figs. S1-S3).**

| **Fig.** | **Task** | **Statistical Test** | **Factor** | **F-Value** | **P-Value** | **Post-Hoc**  **(Displayed if P<0.1)** |
| --- | --- | --- | --- | --- | --- | --- |
| **S1A** | **FR1**  Pellets (#) | Two-Way RM ANOVA | Session × Treatment | F (16, 432) = 1.919 | 0.0173 | Tukey’s Multiple Comparisons test  Session 1 VEH vs. AIR:  P=0.0168 |
|  |  |  | Session | F (8, 432) = 42.95 | <0.0001 |  |
|  |  |  | Treatment | F (2, 54) = 0.3898 | 0.6791 |  |
|  |  |  | Subject | F (54, 432) = 5.588 | <0.0001 |  |
| **S1B** | **FR1**  Inactive Pokes (#) | Two-Way RM ANOVA | Session × Treatment | F (16, 432) = 1.821 | 0.0264 | Tukey’s Multiple Comparisons test  Session 1  VEH vs. AIR:  P=0.0565 |
|  |  |  | Session | F (8, 432) = 0.4087 | 0.9156 |  |
|  |  |  | Treatment | F (2, 54) = 0.03596 | 0.9647 |  |
|  |  |  | Subject | F (54, 432) = 3.702 | <0.0001 |  |
| **S1C** | **FR1**  Latency to First Retrieval (s) | Mixed Effects Model (REML) | Session | F (2.749, 138.5) = 12.47 | <0.0001 | Tukey’s Multiple Comparisons test  Session 2  VEH vs. NIC: P=0.0639  Session 9  AIR vs. VEH: P=0.0639  VEH vs. NIC: P=0.0928 |
|  |  |  | Treatment | F (2, 54) = 6.321 | 0.0034 |  |
|  |  |  | Session × Treatment | F (16, 403) = 0.9894 | 0.4674 |  |
| **S1D** | **FR1**  Average Inter-pellet interval | Mixed Effects Model (REML) | Session | F (4.004, 211.2) = 45.05 | <0.0001 | Tukey’s Multiple Comparisons test  Session 1  AIR vs. VEH: P=0.0112 *  VEH vs. NIC: P=0.0836  Session 8  AIR vs. NIC: P=0.0867 |
|  |  |  | Treatment | F (2, 54) = 1.533 | 0.2252 |  |
|  |  |  | Session × Treatment | F (16, 422) = 2.945 | 0.0001 |  |
|  |  |  |  |  |  |  |
| **S2A** | **PRT**  Active Pokes (#) | Kruskal-Wallis Test | All Groups | Kruskal-Wallis Statistic  H(2) = 7.470 | 0.0239 | Dunn’s Multiple Comparisons test  AIR vs. VEH: P=0.0222 * |
| **S2B** | **PRT**  Inactive Pokes (#) | Kruskal-Wallis Test | All Groups | Kruskal-Wallis Statistic  H(2) = 11.52 | 0.0031 | Dunn’s Multiple Comparisons test  AIR vs. VEH: P=0.0021 ** |
| **S2C** | **PRT**  Pellets (#) | Kruskal-Wallis Test | All Groups | Kruskal-Wallis Statistic  H(2) = 7.609 | 0.0223 | Dunn’s Multiple Comparisons test  AIR vs. VEH: P=0.0208 * |
| **S2D** | **PRT**  Latency to First Retrieval (s) | Kruskal-Wallis Test | All Groups | Kruskal-Wallis Statistic  H(2) = 8.448 | 0.0146 | Dunn’s Multiple Comparisons test  AIR vs. VEH: P=0.0874  VEH vs. NIC: P=0.0177 * |
| **S2E** | **PRT**  Average Inter-pellet Interval (s) | Kruskal-Wallis Test | All Groups | Kruskal-Wallis Statistic  H(2) = 7.083 | 0.0290 | Dunn’s Multiple Comparisons test  AIR vs. NIC: P=0.0256 * |
| **S2F** | **PRT**  Average Pellet retrieval Time (s) | Kruskal-Wallis Test | All Groups | Kruskal-Wallis Statistic  H(2) = 3.373 | 0.1852 |  |
| **S3A** | **QT**  Latency to First Retrieval (s) | Kruskal-Wallis Test | All Groups | Kruskal-Wallis Statistic  H(2) = 14.90 | 0.0006 | Dunn’s Multiple Comparisons test  AIR vs. VEH: P=0.0005 ***  VEH vs. NIC: P=0.0319 * |
| **S3B** | **QT**  Average Inter-pellet Interval (s) | Kruskal-Wallis Test | All Groups | Kruskal-Wallis Statistic  H(2) = 2.722 | 0.2564 |  |
| **S3C** | **QT**  Average Pellet retrieval Time (s) | Kruskal-Wallis Test | All Groups | Kruskal-Wallis Statistic  H(2) = 3.286 | 0.1934 |  |
| **S3D** | **QT**  Pellets Consumed | Two-Way RM ANOVA | Treatment x Session Type | F (2, 52) = 6.509 | 0.0030 | Bonferroni’s Multiple Comparisons test  SUCROSE-QUININE  AIR: P=0.0036 **  VEH: P=0.0007 *** |
|  |  |  | Treatment | F (2, 52) = 7.248 | 0.0017 |  |
|  |  |  | Session Type | F (1, 52) = 16.19 | 0.0002 |  |
|  |  |  | Subject | F (52, 52) = 1.039 | 0.4460 |  |
